# ChatGPT’s role in alleviating anxiety in total knee arthroplasty consent process: a randomized controlled trial pilot study

**DOI:** 10.1097/JS9.0000000000002223

**Published:** 2025-02-04

**Authors:** Wenyi Gan, Jianfeng Ouyang, Guorong She, Zhaowen Xue, Lingxuan Zhu, Anqi Lin, Weiming Mou, Aimin Jiang, Chang Qi, Quan Cheng, Peng Luo, Hua Li, Xiaofei Zheng

**Affiliations:** a Department of Joint Surgery and Sports Medicine, Zhuhai People’s Hospital (The Affiliated Hospital of Beijing Institute of Technology, Zhuhai Clinical Medical College of Jinan University), Zhuhai, Guangdong, China; b Department of Bone and Joint Surgery and Sports Medicine Center, The First Affiliated Hospital, The First Affiliated Hospital of Jinan University, Guangzhou, China; c Department of Oncology, Zhujiang Hospital, Southern Medical University, Guangzhou, Guangdong, China; d Department of Urology, Shanghai General Hospital, Shanghai Jiao Tong University School of Medicine, Shanghai, China; e Department of Urology, Changhai hospital, Naval Medical University (Second Military Medical University), Shanghai, China; f The University of Hong Kong, Hong Kong, China; g Department of Neurosurgery, Xiangya Hospital, Central South University, Changsha, Hunan, China; h Department of Foot and Ankle Surgery, Beijing Jishuitan Hospital, Capital Medical University, Beijing, China

**Keywords:** ChatGPT, informed consent, patient education, perioperative satisfaction, psychological evaluation, total knee arthroplasty

## Abstract

**Background:**

Recent advancements in artificial intelligence (AI) like ChatGPT have expanded possibilities for patient education, yet its impact on perioperative anxiety in total knee arthroplasty (TKA) patients remains unexplored.

**Methods:**

In this single-blind, randomized controlled pilot study from April to July 2023, 60 patients were randomly allocated using sealed envelopes to either ChatGPT-assisted or traditional surgeon-led informed consent groups. In the ChatGPT group, physicians used ChatGPT 4.0 to provide standardized, comprehensive responses to patient queries during the consent process, while maintaining their role in interpreting and contextualizing the information. Outcomes were measured using Hospital Anxiety and Depression Scales (HADS), Perioperative Apprehension Scale-7 (PAS-7), Visual Analogue Scales for Anxiety and Pain (VAS-A, VAS-P), Western Ontario and McMaster Universities Osteoarthritis Index (WOMAC), and satisfaction questionnaires.

**Results:**

Of 55 patients completing the study, the ChatGPT group showed significantly lower anxiety scores after informed consent (HADS-A: 10.48 ± 3.84 vs 12.75 ± 4.12, *P* = .04, Power = .67; PAS-7: 12.44 ± 3.70 vs 14.64 ± 2.11, *P* = .01, Power = .85; VAS-A: 5.40 ± 1.89 vs 6.71 ± 2.27, *P* = .02, Power = .75) and on the fifth postoperative day (HADS-A: 8.33 ± 3.20 vs 10.71 ± 3.83, *P* = .01, Power = .79; VAS-A: 3.41 ± 1.58 vs 4.64 ± 1.70, *P* = .008, Power = .85). The ChatGPT group also reported higher satisfaction with preoperative education (4.22 ± 0.51 vs 3.43 ± 0.84, *P*<.001, Power = .99) and overall hospitalization experience (4.11 ± 0.65 vs 3.46 ± 0.69, *P* = .001, Power = .97). No significant differences were found in depression scores, knee function, or pain levels.

**Conclusions:**

ChatGPT-assisted informed consent effectively reduced perioperative anxiety and improved patient satisfaction in TKA patients. While these preliminary findings are promising, larger studies are needed to validate these results and explore broader applications of AI in preoperative patient education.

## Introduction

**Highlights**

- In our randomized controlled pilot study, we found that using ChatGPT to assist with the informed consent process before total knee arthroplasty significantly reduced patients’ perioperative anxiety levels and improved their satisfaction with preoperative education and overall hospital experience.
- We had three independent orthopedic surgeons evaluate ChatGPT’s responses to the 10 most common patient questions, and they deemed the responses excellent in terms of accuracy, completeness, objectivity, and acceptability. This suggests that ChatGPT can be a beneficial supplement to the preoperative informed consent process.
- Although ChatGPT cannot replace physicians in making clinical decisions, our study found that it benefits both physicians and patients in doctor-patient communication. Physicians can interpret and supplement ChatGPT’s responses based on their own clinical experience to provide more personalized answers to patients. At the same time, patients’ need to search online is reduced, and their trust in physicians is enhanced with the support of objective evidence from AI.

The exchange of information between patients and physicians before surgery is crucial for building confidence between the two parties^[1]^. During the informed consent process, doctors should clearly explain the disease’s cause, progression, and treatment options, detail the stages of treatment along with benefits and surgical risks, and thoroughly address any patient questions^[2,3]^. This may be due to communication barriers caused by unequal knowledge bases between doctors and patients, leading to inconsistencies in understanding treatment plans and expectations regarding disease outcomes^[4,5]^. Studies reveal that most of respondents use social media for health information, but less than a third check the reliability of these sources, indicating that internet advancements have not significantly altered doctor–patient communication^[6-9]^. The relationship between physicians and patients is growing more contentious^[10,11]^, leading to an increase in legal disputes, as standardized informed consent processes often neglect personalized responses to patient inquiries, with some doctors relying on department-prepared materials to address common questions^[6]^. However, patients perceive these answers as subjective, and the source of educational materials lacks transparency, leaving no objective way to alleviate their concerns^[12-14]^. With the rapid development of the Internet and rise of content creators, online information about various types of surgery tends to emphasize the benefits while disregarding the risks^[3,15]^. Although numerous studies have been conducted regarding the use of video display and animation demonstration to improve patients’ understanding of diseases and surgical procedures, such methods are not interactive and thus cannot objectively answer patients’ specific questions^[16,17]^. The American College of Surgeons believes that a standardized, effective informed consent process could lead to better patient outcomes and fewer medical disputes^[18]^.

As a large language model (LLM), ChatGPT offers unique opportunities in the informed consent process by balancing the standardization and personalization of patient education materials and ensuring the transparency of their sources^[19,20]^. As an artificial intelligence (AI) based conversational model, ChatGPT can promptly respond to various medical queries^[19,20]^. It has proven to provide accurate and consistent answers across multiple disciplines such as ophthalmology, otolaryngology, gynecology, and gastroenterology, showcasing its extensive multidisciplinary medical knowledge base^[21-24]^. It can even pass professional exams in various countries and fields, including orthopedics^[25]^. Additionally, ChatGPT has demonstrated its capability to deliver precise responses and explain potential surgical complications^[26-28]^ during the perioperative periods of thoracic surgery^[26]^, robot-assisted radical prostatectomy^[27]^, and joint replacement surgery^[28]^. While some researchers suggest that ChatGPT could enhance patient satisfaction and reduce perioperative anxiety^[26-28]^, a primary concern arises when patients directly use it: their ability to evaluate and clarify AI-generated content^[29]^. LLMs have demonstrated proven capability in making complex medical information more accessible to non-professionals through text refinement and readability enhancement, with cross-sectional studies validating this advantage in preoperative informed consent processes^[30]^. Despite these promising applications, higher-level evidence from randomized controlled trials is still needed to fully validate ChatGPT’s impact on perioperative patient education.

Total knee arthroplasty (TKA) is the most common and effective surgical treatment for terminal osteoarthritis (OA)^[31]^. With successful surgery, patients can regain nearly normal knee function, but complications such as postoperative pain, rigidity, and infection can result in multiple surgeries and even mortality. OA is a common chronic disease in the elderly, who have a strong desire for treatment but who also are uncertain about procedures such as implant, joint amputation, and reconstruction of active metal prosthesis^[32,33]^. Such anxiety can reduce treatment efficacy as well as the patient’s medical compliance^[33,34]^.

Therefore, we initiated a single-blind, prospective, randomized, controlled pilot study with a relatively small sample size to preliminarily assess the potential efficacy of ChatGPT in enhancing the process of informed consent for TKA. This pioneering study aimed to explore whether the utilization of ChatGPT could mitigate patients’ anxiety during hospitalization, augment their understanding of the disease, and improve their satisfaction with the treatment process, thereby laying a robust foundation for future large-scale clinical trials.

## Method

### Study design

This was a prospective, randomized, single-blind, controlled clinical pilot study. In accordance with the Declaration of Helsinki, all participant information was collected after obtaining written informed consent. Participants were included in the results only if they successfully completed the surgery and all measures concurrently. We used the CONSORT protocol, a flow diagram of which is available in Supplemental Digital Content 1 (http://links.lww.com/JS9/D748)^[35]^. Additionally, we have incorporated the CONSORT-EHEALTH^[36]^ in Supplemental Digital Content 2 (http://links.lww.com/JS9/D749).

### Participants

**Figure 1.**
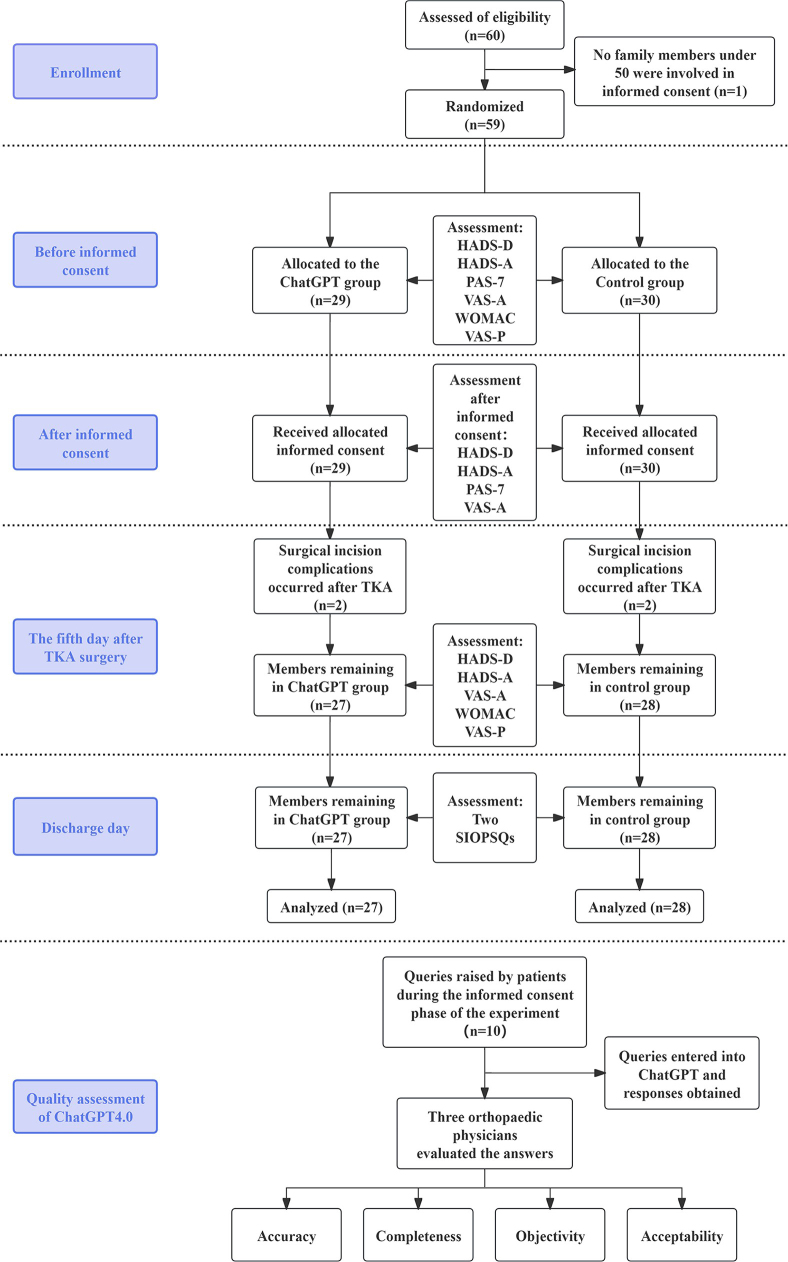
Enhanced and expanded flowchart, based on CONSORT 2010, depicting the entire investigation process. TKA: total knee arthroplasty; HADS-A: hospital anxiety and depression scales for anxiety; HADS-D: hospital anxiety and depression scales for depression; PAS-7: perioperative apprehension scale-7; VAS-A: visual analogue scale for Anxiety; WOMAC: Western Ontario and McMaster Universities Osteoarthritis Index; VAS-P: visual analogue scale for pain; SIOPSQs: single-item overall patient satisfaction questionnaires.

Study participants were patients with knee OA who were admitted to the clinic of the Bone, Joint, and Sports Medicine Centre at the The First Affiliated Hospital of Jinan University University. Patients who met the inclusion criteria were randomized into ChatGPT and control groups, and demographic information was collected, as listed in Table 1. Relevant scales were assessed at various times before admittance, the night before surgery (after signing informed consent), 5 days after surgery, and at discharge (Fig. 1).

**Table 1:** Baseline patient characteristics

Characteristics	ChatGPT group (*n* = 27)	Control group (*n* = 28)	Total (*n* = 55)	*P* value
Age (mean ± SD, range)	72.37 ± 4.27 (61–80)	73.04 ± 5.21 (60–80)	72.71 ± 4.74 (60–80)	.505
Gender (male:female)	5:22	7:21	12:43	.561
Operative site (left:right)	17:10	20:8	37:18	.504
Educational level (≥bachelor degree: < bachelor degree: no educational experience)	10:14:3	8:16:4	18:30:7	.786
HADS-D (mean ± SD)	13.81 ± 4.31	13.68 ± 4.54	13.75 ± 4.39	.859
HADS-A (mean ± SD)	13.52 ± 4.62	13.43 ± 4.81	13.47 ± 4.67	.953
PAS-7 (mean ± SD)	14.44 ± 3.24	14.54 ± 3.17	14.49 ± 3.17	.916
VAS-A (mean ± SD)	6.74 ± 2.10	6.57 ± 2.30	6.65 ± 2.19	.822
WOMAC (mean ± SD)	109.93 ± 13.98	111.18 ± 12.12	110.56 ± 12.96	.840
VAS-P (mean ± SD)	6.15 ± 1.83	6.07 ± 1.92	6.11 ± 1.86	.864

#### Inclusion criteria

1) Age: 45–80 years.

2) Diagnosis: Knee OA with recurrent knee pain over the prior month.

3) Symptoms: Average morning stiffness <30 minutes, occasional bony fricative sounds.

4) Radiographic Criteria: Kellgren–Lawrence grade IV on preoperative full-length standing X-rays.

5) Insurance: Covered by employee or resident medical insurance.

6) Family Support: Family member under 50 years present during preoperative consent.

#### Exclusion criteria

1) Mental health: Presence of mental disorders.

2) Other pain: Primary complaint of hip or low back pain, or history of total hip replacement.

3) Surgical history: Prior knee collateral ligament reconstruction or osteotomy of distal femur or proximal tibia.

4) Physical condition: Body mass index >30 kg/m^2^, muscle strength < grade III, wheelchair use > 3 months.

5) Neuromuscular diseases: Sequelae of poliomyelitis, Parkinson’s disease.

6) Cognitive impairment: Alzheimer’s disease or brain atrophy.

7)Joint conditions: Charcot’s joint, severe varus (>30°) or valgus deformities, extra-articular deformity (>10°).

8)Postoperative issues: Complications like aseptic prosthesis displacement, periprosthetic fracture, infection, or nonunion/necrosis of incision.

### Perioperative management

All patients enrolled in this study completed the diagnosis and treatment process under the care of two treatment groups. The decision for TKA surgery in OA patients was made jointly by the two primary surgeons from each treatment group. Furthermore, during the TKA procedures throughout the study period, the primary surgeons from each group served as the first assistant for the other group’s TKA surgeries.

Before surgery, anemia and hypoproteinemia were corrected. Celecoxib 200 mg was administered orally the night before surgery for preemptive analgesia. Two to three hours before surgery, patients received 200 mL of oral enteral nutrition powder. Neither intravascular catheters nor drainage tubes were placed during or after the operation. Local and intermittent cold therapy was applied to the affected knee within 48 hours after surgery. We used brochures and multimedia materials to educate patients and their families about measures for rapid postoperative recovery. A rehabilitation therapist instructed patients in postoperative functional exercises and the use of mobility aids. After recovery from anesthesia, patients began ankle pump exercises, quadriceps isometric contractions, active knee flexion and extension, and straight leg raises. Within 24 hours after surgery, patients began walking short distances with full weight-bearing using a walker. All participants received the same surgical anesthesia and postoperative analgesia protocol.

### Intervention

During preoperative interviews, patients in both groups were given the “TKA Publicity Manual and Typical Cases,” which contains information about the surgical procedure, prosthesis type, typical cases in the department, recent follow-up, and long-term outcomes. Table 2 presents details on other aspects of the informed consent process and how they were provided. Implementer A supervised the informed consent process, which included tracking patient group assignments and recording the questions raised by the patients. Implementers B and C managed the use of ChatGPT software during the acquisition of informed consent for patients in the ChatGPT group.

**Table 2:** Summary of interventions provided to each group

	ChatGPT group	Control group
Perioperative education on admission	✓	✓
Informed consent assisted by department education materials	✓	
Informed consent with ChatGPT assistance	✓	✓
Performing unilateral TKA surgery	✓	✓
ERAS strategy used in perioperative period	✓	✓

### Control group: Traditional informed consent

**Figure 2.**
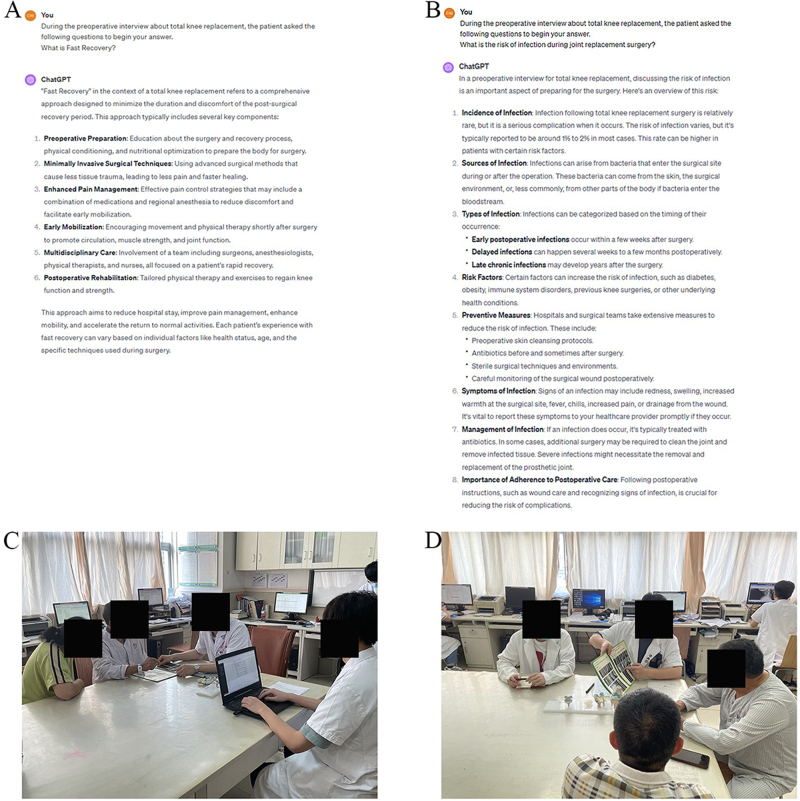
Preoperative informed consent process. (A, B) Two examples of ChatGPT4.0 (Chinese version) responding to patient questions; (C) Patients in the ChatGPT group receiving preoperative informed consent; (D) Patients in the control group receiving preoperative informed consent.

In the control group, the surgeon first explained the patient’s condition and the necessity of TKA surgery based on the results of the physical examination and imaging. Then, based on preoperative interview data, he described the process of TKA surgery and the postoperative prognosis. Finally, he orally answered questions from patients and their families. Once they and their families had no further questions about the treatment plan, patients were instructed to submit the form indicating informed consent for surgery. Fig. 2D illustrates the informed consent process with the control group.

### ChatGPT group: Informed consent assisted by ChatGPT

In the ChatGPT group, the consultation began as with the control group: the physician detailed the patient’s condition and the need for TKA surgery. ChatGPT was then introduced to supplement the discussion with additional, real-time information and to respond to patients’ queries. The physician entered each query into the ChatGPT interface, critically evaluating the AI’s responses and integrating third-party data to ensure accuracy and objectivity. This approach not only provided verified information but also allowed for personalization of the communication based on the patient’s specific needs^[37,38]^. The physician’s role in mediating this information reinforced the thoroughness of the consultation. Patients and families, once they had no further questions, were guided to complete the informed consent forms. Fig. 2A and B illustrate examples of patient queries and ChatGPT responses, and Figure 2C shows the informed consent process, highlighting the role of ChatGPT.

### Choosing ChatGPT model and setting parameters

We accessed the ChatGPT 4.0 model through a website^[39]^ (https://chat.openai.com/?model=gpt-4) instead of connecting to OpenAI’s API via a local workstation. Thus, we utilized the standard version of ChatGPT 4.0 without any customized parameter settings. We did not use any of GPT-4’s built-in plugins and we disabled the chat history & training feature. The temperature parameter was set to the default of 0.7, and we used the default of 0.9 for the Top-p parameter. To minimize the potential influence of one question on another and maintain the independence of each GPT-4 response, we initiated a new chat for every question posed by patients.

### Assessments

Assessments included three psychological assessment instruments, one knee function scale, one pain score, and two questionnaires of the patient’s overall satisfaction with their education and hospital experience (Fig. 1).

The Hospital Anxiety and Depression Scale (HADS) is a self-rating scale created in 1983 by A S Zigmond and R P Snaith to assess the emotional states of hospitalized patients^[40]^. It consists of subscales for anxiety (HADS-A) and depression (HADS-D). The Perioperative Apprehension Scale-7 (PAS-7) is a Chinese population-based self-rating scale to assess patients’ apprehension before surgery^[41]^. The anxiety level of patients was evaluated with seven items within the dimensions of mental and physical anxiety. The Visual Analogue Scale for Anxiety (VAS-A), implemented in 1976, is a fast and effective scoring method with which patients rate their own anxiety on a scale from 1 to 10, with larger scores indicating greater anxiety^[42]^.

The Western Ontario and McMaster Universities OA Index (WOMAC) is a valid and dependable disease-specific measure of knee OA pain, rigidity, and physical function^[43]^. A higher WOMAC subscale score indicates more severe symptoms or functional limitations. It is used to evaluate the severity of the arthritis and/or the efficacy of the treatment. The visual analogue scale for pain (VAS-P) is a comprehensive self-evaluation of pain based primarily on the patient’s sensation of pain in the afflicted limb during daily activities^[44]^. The VAS-P score ranges from 0 to 10, with higher scores indicating a more intense pain sensation.

On the first day of hospitalization, HADS, PAS-7, VAS-A, WOMAC, and VAS-P were administered to the patients in this study. The HADS, PAS-7, and VAS-A were evaluated after patients provided informed consent prior to surgery. The HADS, VAS-A, WOMAC, and VAS-P were assessed again on the fifth postoperative day. On the day of the patient’s discharge, we administered two Single-Item Overall Patient Satisfaction Questionnaires (SIOPSQs), consisting of a single query each, to assess the patients’ comprehension of the TKA and satisfaction with their overall medical experience. Patients were asked to rate their experience on a scale of 1 to 5, with 1 representing very dissatisfied, and 5, very satisfied.

Finally, we analyzed the most frequent queries (Supplemental Digital Content 3, http://links.lww.com/JS9/D750) raised by patients in both the ChatGPT group and the control group during the informed consent phase of the experiment, with implementer A responsible for tallying the frequency of these queries. To evaluate ChatGPT’s responses, we compiled the ten most common questions, entered them into ChatGPT4.0, and captured screenshots of ChatGPT’s answers. Three independent orthopedic surgeons evaluated the accuracy, completeness, objectivity, and acceptability of the responses without knowing their source. The questions and evaluations are listed in Table 3.

**Table 3:** Evaluation of ChatGPT4.0 responses to the 10 most-asked patient queries during the informed consent process

	Accuracy	Completeness	Objectivity	Acceptance
What is ERAS? (Frequency: 23)				
Average scores (*n* = 3)	5.00	5.00	5.00	5.00
What is the most effective remedy for my condition? Can minimally invasive arthroscopic surgery rectify my condition? (Frequency: 18)				
Average scores (*n* = 3)	5.00	5.00	5.00	5.00
What is the artificial joint composed of? (Frequency: 39)				
Average scores (*n* = 3)	5.00	4.00	5.00	5.00
I’ve heard that functional exercise following joint surgery is extremely excruciating. Is there any method to alleviate the discomfort? (Frequency: 17)				
Average scores (*n* = 3)	5.00	4.33	5.00	5.00
How long will it be before I can move and walk following surgery? (Frequency: 49)				
Average scores (*n* = 3)	5.00	4.00	5.00	5.00
Surgical procedures pose a risk of vascular and nerve injury; which of these risks is specific? The potential for damage? What are the repercussions? (Frequency: 31)				
Average scores (*n* = 3)	5.00	5.00	5.00	5.00
What is the infection risk associated with TKA surgery? (Frequency: 41)				
Average scores (*n* = 3)	5.00	5.00	5.00	5.00
What is the risk of developing venous thrombosis during total joint replacement? What is required? (Frequency: 11)				
Average scores (*n* = 3)	5.00	4.66	5.00	5.00
How long do artificial joints typically last? (Frequency: 50)				
Average scores (*n* = 3)	5.00	5.00	5.00	5.00
Are there any issues to watch out for or things to prevent following artificial joint surgery? (Frequency: 48)				
Average scores (*n* = 3)	5.00	5.00	5.00	5.00
1 = Very dissatisfied; 2 = Dissatisfied; 3 = Neutral; 4 = Satisfied; 5 = Very satisfied				

### Blinding

In our study, patients, project implementers B and C, and the physicians conducting preoperative interviews were informed of the study’s classification. Project implementers B and C collaborated with physicians in the ChatGPT group to ensure that questions were entered into ChatGPT in a way that remained consistent with the core of the patients’ inquiries, without the need for specialized training in operating ChatGPT. Primary physicians first listened to patient’s personalized questions and then refined these inquiries to 20 words or less. Then, implementers B and C entered the refined questions into ChatGPT for responses.

To minimize systematic error and human bias, key personnel, including the chief surgeon in charge of the operation, the attending nurse, the postoperative rehabilitation therapist, and researcher A, who collected the study’s observational data, were blinded to patient group assignments until the end of this clinical pilot study, ensuring impartiality in treatment and data collection.

### Randomization

To ensure an equal distribution of patients into the two groups and maintain consistent numbers within each group, we utilized the sealed envelope method, with 30 envelopes designated for each group: “traditional informed consent” and “ChatGPT-assisted informed consent.” This approach served as a method of blocking to achieve balanced group sizes. A nurse not involved in the study opened an envelope for each patient enrolled in the experiment on the second-day post-hospitalization and informed project implementers B and C of the group assignments, thus preserving the integrity of the randomization.

### Statistics

To assess statistical power, we first calculated Cohen’s *d* to quantify the difference between the means of two independent samples^[45]^. The specific method used was to standardize the expected mean difference using pooled standard deviation. Subsequently, we conducted a post-hoc power analysis using G*Power software^[46]^, with input parameters including sample sizes, a one-tailed test, and an alpha error probability set at 0.05. This step helped us determine our experiment’s capability to detect actual effects given the specified sample sizes and effect size.

SPSS software version 26.0 was used for statistical analysis. We used Chi-square analysis to examine gender disparities between groupings and the Shapiro-Wilk test to determine whether the data exhibited a normal distribution. If the data for this index did not follow a normal distribution, we analyzed it with the Mann–Whitney test. Aside from that, the data were processed based on Levene’s test, and it was determined that the variance was homogeneous; thus, we performed an independent sample T-test. A *P* value of less than .05 was considered statistically significant. Fleiss’ kappa, serving as a generalization of this statistic, was used in SPSS to evaluate the consistency among the three raters for the ChatGPT response qualities of “Accuracy,” “Completeness,” “Objectivity,” and “Acceptance.” We generated bar charts from a portion of the data with GraphPad Prism 8.

### Ethics approval

This study was approved by the XXX Hospital of XXX University’s ethics committee KY-XXX), and it was registered in the Chinese Clinical Trials Registry (XXX). To view experiments pertaining to the registration process and its associated accessories, refer to the following URL: https://www.chictr.org.cn/hvshowproject.html?id=242226&v=1.0. The website attachment details the compensation and reward structure for participants as well as the measures taken to protect their privacy following the conclusion of the experiment. To request access to the original data, email the corresponding author to ensure the confidentiality of participants.

## Results

From April to July 2023, a study enrolled 60 TKA patients. Due to postoperative complications and consent issues, five patients withdrew or were excluded: three from the ChatGPT group (two for incision complications, one for consent issues) and two from the control group (both for incision complications) (Fig. 1). Ultimately, 55 patients completed the study, having met all inclusion criteria including informed consent and necessary assessments.

Demographically, the ChatGPT group consisted of 27 patients (average age 72.37 ± 4.27, 5 men and 22 women, 10 right knee and 17 left knee TKAs, education distribution: 10 with a bachelor’s degree or higher, 14 with less, 3 with none) (Table 1). The control group included 28 patients (average age 73.04 ± 5.21, 7 men and 21 women, 8 right knee and 20 left knee TKAs, education distribution: 8 with a bachelor’s degree or higher, 16 with less, 4 with none) (Table 1). No significant differences were found between groups in terms of age, gender ratio, knee joint ratio, education level (Table 1), or initial measurements of HADS-D, HADS-A, PAS7, VAS-A, WOMAC, and VAS-P (Supplemental Digital Content 4, http://links.lww.com/JS9/D751).

### Psychological anxiety and depression evaluation during hospitalization

**Figure 3.**
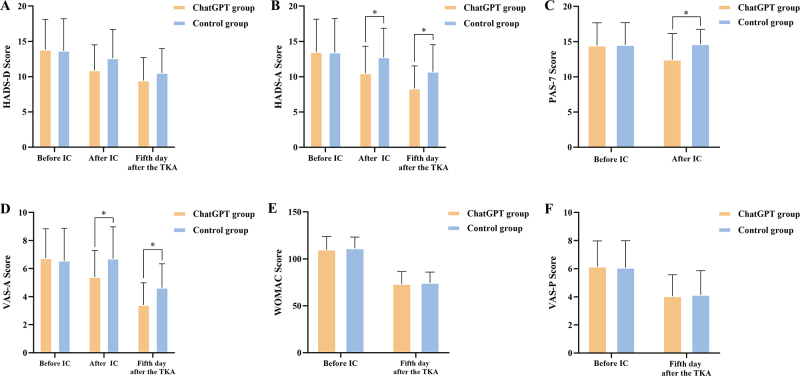
Comparison of assessment scores administered at various times to the ChatGPT group and control group. (A) HADS-D scores; (B) HADS-A scores; (C) PAS-7 scores; (D) VAS-A scores; (E) WOMAC scores; (F) VAS-P scores. HADS-A: hospital anxiety and depression scales for anxiety; HADS-D: hospital anxiety and depression scales for depression; PAS-7: perioperative apprehension scale-7; VAS-A: visual analogue scale for Anxiety; WOMAC: Western Ontario and McMaster Universities Osteoarthritis Index; VAS-P: visual analogue scale for pain.

Figure 3 presents a comparison of assessment scores between groups. The use of ChatGPT during informed consent significantly reduced preoperative HADS-A (10.48 ± 3.84 vs. 12.75 ± 4.12, *P* = .04, Power = .67) and PAS-7 (12.44 ± 3.70 vs. 14.64 ± 2.11, *P* = .01, Power = .85) scores in the experimental group, with strong statistical power supporting these findings. The VAS-A scores for apprehension also favored the experimental group (5.40 ± 1.89 vs. 6.71 ± 2.27, *P* = .02, Power = .75). Furthermore, on the fifth postoperative day, the experimental group reported less anxiety on both the HADS-A (8.33 ± 3.20 vs 10.71 ± 3.83, *P* = .01, Power = .79) and VAS-A (3.41 ± 1.58 vs 4.64 ± 1.70, *P* = .008, Power = .85), with significant power indicating reliable results. However, the differences in HADS-D scores both preoperatively (10.89 ± 3.61 vs 12.61 ± 4.07, *P* = .10, Power = .50) and postoperatively (9.44 ± 3.26 vs 10.54 ± 3.45, *P* = .22, Power = .33) were not significant, likely due to insufficient power.

**Figure 4.**
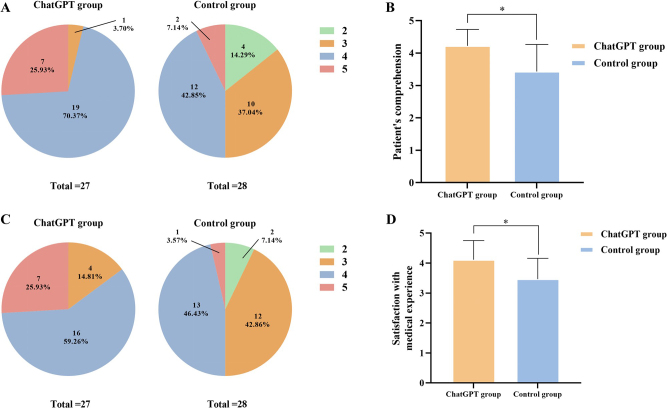
Distribution and comparison of overall hospitalization satisfaction and educational satisfaction ratings. (A, B) Comparison and distribution of educational satisfaction scores between the ChatGPT group and the control group; (C, D) Comparison and distribution of hospitalization satisfaction scores between the ChatGPT group and the control group.

### Evaluation of knee function and discomfort during hospitalization

On the fifth day following TKA, the experimental group showed a tendency toward better knee function and slightly less pain than the control group according to WOMAC (73.33 ± 13.22 vs 74.43 ± 11.57, *P* = .75, Power = .09) and VAS-P scores (4.04 ± 1.53 vs 4.14 ± 1.72, *P* = .88, Power = .08), but these minor differences were not statistically significant and are considered clinically negligible (Fig. 3).

### Assessment of satisfaction with education and hospitalization

On discharge day, the experimental group rated their preoperative education (4.22 ± 0.51 vs 3.43 ± 0.84, *P*<.001, Power = .99) and hospitalization experience (4.11 ± 0.65 vs 3.46 ± 0.69, *P* = .001, Power = .97) more favorably than the control group, with these significant differences supported by strong statistical power, suggesting these results are robust (Fig. 4).

### Assessment of ChatGPT response caliber

Three independent physicians unanimously rated ChatGPT’s responses to 10 questions as flawless in accuracy, objectivity, and acceptability, each scoring a perfect 5. In terms of completeness, ratings included 21 “very satisfied” and 9 “satisfied” (Table 3). The Fleiss’ kappa index for completeness was 0.68, indicating substantial agreement among the raters. Due to the unanimity in scoring for accuracy, objectivity, and acceptance, Fleiss’ kappa was considered irrelevant for these criteria, reflecting strong consensus (Supplemental Digital Content 5, http://links.lww.com/JS9/D752).

## Discussion

This single-blind, randomized, controlled pilot study assessed the efficacy of using ChatGPT to enhance the informed consent process before TKA. Findings revealed that ChatGPT-assisted patients experienced significantly reduced perioperative anxiety, better understanding of knee OA, and greater hospital satisfaction compared to the control group, with post hoc analysis showing power values close to or exceeding 0.8 for these outcomes. Although differences in knee function recovery and pain were not statistically significant, likely due to the limited sample size of the pilot study, these results suggest a need for further large-scale, multicenter studies to confirm these findings and fully assess the benefits of ChatGPT in clinical settings. Expert evaluations of ChatGPT’s responses were consistently high across multiple dimensions, indicating strong agreement among specialists regarding the quality and relevance of the information provided. The study supports ChatGPT’s utility in improving patient care through personalized and informed consent processes, particularly in enhancing psychological well-being.

Knee OA, as a chronic condition, typically imposes long-term psychological and financial burdens on patients^[47]^. Before undergoing TKA, patients may have endured years of pain and limited mobility, leading not only to gradual decline in overall physical function but also severely impacting their quality of life^[47,48]^. In such circumstances, patients often turn to the Internet for relevant information^[49]^; however, lacking professional medical knowledge, they struggle to accurately evaluate the reliability of this information, potentially exacerbating their anxiety and depression^[50,51]^. Studies have shown that patient anxiety and depression are significant risk factors affecting treatment outcomes and long-term prognosis^[52]^. While TKA remains the most effective treatment for end-stage knee OA^[53]^, preoperative psychological assessment has not yet become a routine component of preoperative evaluation^[54]^. Notably, the “white coat effect” commonly present during preoperative informed consent can cause patients to experience tension and anxiety when facing medical staff, not only affecting effective doctor-patient communication but potentially reducing patients’ comprehension and acceptance of disease and surgery-related information^[55]^. Currently, the primary approach to alleviating preoperative anxiety relies on doctor-patient communication during the informed consent process, and research indicates that more severe preoperative anxiety/depression symptoms correlate with poorer long-term TKA outcomes^[56]^. Therefore, a standardized and effective informed consent process should focus on conveying accurate and reliable information, effectively addressing patients’ long-standing questions to alleviate anxiety, and helping patients develop appropriate expectations regarding surgical outcomes, thereby establishing a solid psychological foundation for surgical treatment^[53]^.

The informed consent form is an essential component of the preoperative consultation, the most crucial dialogue between physicians and patients in surgery departments, and a vital link in the implementation of patients’ right to know^[15,57,58]^. The two primary components of informed consent are standard medical education for patients and individualized responses to patients’ queries. Good informed consent can increase communication and trust between physicians and patients, reduce doctor–patient conflicts and potential medical litigation, and improve the mood of patients during hospitalization^[59]^. While current researchers have predominantly focused on utilizing multimedia approaches, including both local and web-based multimedia platforms, to enhance patients’ understanding of treatment plans and surgical procedures, these methods have notable limitations^[59,60]^. The multimedia educational tools remain standardized processes that inadequately address individualized patient inquiries. Moreover, they fundamentally maintain a unidirectional flow of information from physician to patient, lacking third-party intervention that could help bridge the knowledge gap and balance the inherent inequalities in doctor-patient communication. This approach fails to address the fundamental disparity in medical knowledge and the power imbalance inherent in doctor-patient dialogues^[61]^.

The development of LLMs presents a unique opportunity to standardize and personalize the preoperative informed consent process. Extensive research has demonstrated the advantages and accuracy of LLMs in various medical consulting applications, showing that they can provide more accurate and comprehensive information than commonly used search engines like Google^[62]^ or community advice platforms like Reddit^[63]^. Despite their benefits, studies also reveal the limitations of LLMs in answering medical questions, particularly in specific areas such as radiology^[29]^. These findings highlight that LLMs should not be primarily used by patients for health consultations; instead, they should function as tools to aid doctors in delivering personalized responses to patient inquiries. When used by physicians, LLMs enhance the transparency of the sources of patient education materials and showcase the professionalism and authority of doctors in verifying the accuracy and clarifying the responses provided by LLMs^[37,64]^. Additionally, the text generation capabilities of LLMs can compensate for the variability in language proficiency among clinicians^[30]^, enabling the creation of more understandable and less jargon-laden educational materials under specific instructions, thereby facilitating more effective patient education^[65]^.

As to our study, we explored the potential clinical applications of ChatGPT, particularly its role in facilitating doctor-patient communication during the informed consent process. The experimental group using ChatGPT demonstrated significantly lower preoperative anxiety scores. However, our findings necessitate discussion from two crucial perspectives: the physicians’ viewpoint and the patients’ perspective. From the physicians’ perspective, while ChatGPT showed promise in facilitating the informed consent process, our study did not fully account for the time investment required to implement LLMs. This includes the time needed to train medical staff in standardized use of these tools and the additional time required to introduce ChatGPT to patients. Furthermore, important ethical considerations arise from utilizing ChatGPT across various clinical scenarios, particularly regarding patient data privacy and the accuracy and appropriateness of AI-generated content. These factors warrant further investigation and resolution in future research. From the patients’ perspective, although ChatGPT-assisted informed consent effectively reduced perioperative anxiety, this pilot study with its limited sample size did not observe significant differences in knee function and postoperative pain outcomes. This lack of significant findings may be attributed to the small sample size. Therefore, whether more definitive results could be obtained through a larger sample size remains to be verified through more extensive subsequent studies.

As science and technology continue to advance, hospitals are poised to innovate in all aspects of clinical diagnosis and treatment, aiming to improve diagnosis rates, treatment efficacy, and patient satisfaction^[57,66,67]^. LLMs like ChatGPT are at the forefront of this innovation, offering significant improvements in clinical accuracy and personalized treatment plans^[68]^. By adeptly processing and analyzing vast amounts of health data, ChatGPT assists healthcare professionals in making rapid and accurate decisions, enhancing both patient outcomes and satisfaction^[68]^. However, this rapid technological advancement also brings challenges such as data privacy, ethical issues, and legal compliance^[69]^. It is crucial to address these concerns through stringent regulations and continuous oversight to ensure that the benefits of such technologies do not come at the expense of patient safety and trust^[70]^. Future research focusing on RCTs will be essential to validate the effectiveness of ChatGPT in clinical applications, helping to refine its use and maximize its benefits in modern medical practices^[71]^.

## Limitations

This study has several limitations, including the lack of long-term follow-up and untracked time costs for the informed consent process. The subjective evaluation of ChatGPT’s answers may have introduced bias, and the exclusion of demographic variations like age, gender, and symptom duration restricts the generalizability of the findings. Additionally, being a single-center pilot study with an insufficient sample size limits the external validity and applicability of the results across different healthcare settings. Despite these issues, the insights gained can guide future research, especially in estimating necessary sample sizes for studies focusing on outcomes like the WOMAC score, tailored to the specific metrics required by varying research objectives. These insights also provide a foundation for subsequent multicenter studies.

## Conclusion

In this randomized, controlled clinical pilot study involving 55 patients with terminal OA, preliminary results suggest that ChatGPT-assisted informed consent may alleviate perioperative anxiety and enhance satisfaction with preoperative education and overall hospital experience. Despite these promising findings, the results are preliminary and were derived from a small, homogeneous sample. Further research in larger, more diverse populations is necessary to confirm these outcomes and explore the broader implications of AI tools like ChatGPT in patient education and care.

## Research registration unique identifying number (UIN)

researchregistry9232.

## Assistance with the study

We sincerely express our gratitude to all of the patients who participated in this clinical pilot study. We are also deeply thankful for the assistance provided by Nurse Gao Zhenghong during our randomization process. Finally, we appreciate Professor Zhenggang Zha and Director Ning Liu for their voluntary involvement in evaluating the quality of ChatGPT’s responses as part of this study. Additionally, Director Ning Liu, alongside Guorong She, assisted in conducting the clinical evaluations and surgeries for all patients.

## Double-blind review policy

As the journal operates a double-blind peer review policy manuscripts should not contain information that would identify the Authors. We changed the relevant information into various number of “XXX.”
